# Sendai Virus-Vectored Vaccines That Express Envelope Glycoproteins of Respiratory Viruses

**DOI:** 10.3390/v13061023

**Published:** 2021-05-29

**Authors:** Charles J. Russell, Julia L. Hurwitz

**Affiliations:** Department of Infectious Diseases, St. Jude Children’s Research Hospital, 262 Danny Thomas Place, Memphis, TN 38105-3678, USA; julia.hurwitz@stjude.org

**Keywords:** vaccine vector, parainfluenza virus, paramyxovirus, pneumovirus, HRSV, envelope glycoprotein, fusion glycoprotein, attachment protein

## Abstract

Human respiratory syncytial virus (HRSV), human metapneumovirus (HMPV), and human parainfluenza viruses (HPIVs) are leading causes of respiratory disease in young children, the elderly, and individuals of all ages with immunosuppression. Vaccination strategies against these pneumoviruses and paramyxoviruses are vast in number, yet no licensed vaccines are available. Here, we review development of Sendai virus (SeV), a versatile pediatric vaccine that can (a) serve as a Jennerian vaccine against HPIV1, (b) serve as a recombinant vaccine against HRSV, HPIV2, HPIV3, and HMPV, (c) accommodate foreign genes for viral glycoproteins in multiple intergenic positions, (d) induce durable, mucosal, B-cell, and T-cell immune responses without enhanced immunopathology, (e) protect cotton rats, African green monkeys, and chimpanzees from infection, and (f) be formulated into a vaccine cocktail. Clinical phase I safety trials of SeV have been completed in adults and 3–6-year-old children. Clinical testing of SeVRSV, an HRSV fusion (F) glycoprotein gene recombinant, has also been completed in adults. Positive results from these studies, and collaborative efforts with the National Institutes of Health and the Serum Institute of India assist advanced development of SeV-based vaccines. Prospects are now good for vaccine successes in infants and consequent protection against serious viral disease.

## 1. The Clinical Need for Vaccines against Human Respiratory Syncytial Virus (HRSV), Human Metapneumovirus (HMPV) and Human Parainfluenza Virus (HPIV)

Acute respiratory tract infections are among the leading causes of death among children and adults worldwide [1]. The ongoing pandemic of coronavirus disease-19 (COVID-19) demonstrates the dire consequences of respiratory infections. Severe acute respiratory syndrome-coronavirus-2 (SARS-CoV-2), the etiologic agent of COVID-19, was first identified in 2019, and has since caused more than 3 million deaths globally [2]. Influenza virus, another well-known respiratory pathogen, contributed to the deaths of more than 50 million people worldwide during the 1918 pandemic [3]. In 2008, seasonal influenza virus caused 28,000 to 111,500 deaths in children less than five years of age [4,5]. In a study by Gaunt et. al. using a world health organization (WHO)-endorsed DALY modeling system, the disease burden of seasonal type A and type B influenza virus was respectively 6.8 and 1.7 per 1000 hospitalized children under the age of five [6].

Less well known are the human respiratory syncytial virus (HRSV), human metapneumovirus (HMPV), and human parainfluenza viruses (HPIV) types 1–4 [7,8,9,10,11,12,13,14,15]. Unfortunately, HRSV, HMPV, and the HPIVs cause considerable morbidity and mortality, particularly among young children and older adults, yet the disease consequences are often falsely attributed to influenza or ‘flu’ infections. In fact, the acute lower respiratory tract infections (ALRTI) caused by HRSV were estimated to kill 55,000–199,000 children under the age of five in 2005, and 94,600–149,400 children under the age of five in 2015 [8,9,10,11,12,13]. In the study described above by Gaunt et. al., the disease burden for HRSV, HMPV, HPIV1, HPIV2, and HPIV3 among hospitalized children under the age of five years was respectively 67.7, 4.0, 0.7, 0.3, and 4.4 per 1000 [6]. It is noteworthy that these estimates, totaling 77.1 per 1000 have far exceeded those for the influenza viruses. For patients who recover from any of the serious respiratory viral infections described above, residual damage to the airway can cause lifelong sequelae [16].

The immune system is capable of controlling infections with these viruses, as is demonstrated by the fact that children who have recovered from a first infection rarely become seriously ill when exposed to the same virus for a second time. Antibodies provide an important first line of defense against re-infection because they can block virus at the point of entry; T cells provide help to other effector cells as well as a fail-safe mechanism by killing infected cells if/when the virus evades the antibody response. Antibodies obtained from adults (pre-immune to HRSV) were once used routinely to passively protect infants from HRSV [17]. More recently, monoclonal antibodies are used to prevent serious infections with HRSV in vulnerable children [18,19,20]. While passive treatments can provide short-term protection, vaccines are needed to induce stable, endogenous virus-specific antibodies and T cells in young infants prior to a first virus exposure. Despite the passing of decades since the first candidate vaccines were developed, there remain no licensed vaccines for HRSV, HMPV, or the HPIVs.

## 2. Virology of the Paramyxoviruses and Pneumoviruses

### 2.1. Taxonomy and Phylogeny

**Figure 1:**
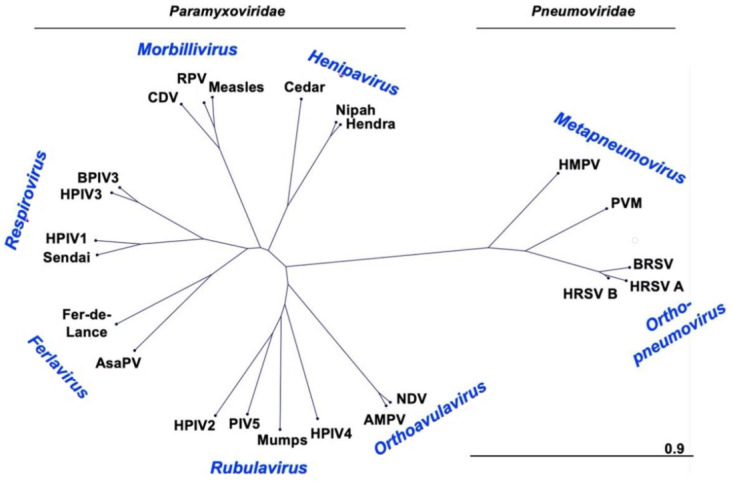
**Phylogenetic tree of F proteins.** Phylogenetic tree was created with CLC Main Workbench Version 20. Scale bar represents branch length as substitutions per site. CDV (canine distemper virus P12569.1), RPV (rinderpest virus P10864.1), Measles (measles virus AAF85680.1), Cedar (Cedar virus AFP87278.1), Nipah (Nipah virus AAM13405.1), Hendra (Hendra virus NP_047111.2), HMPV (ABM67072.1), PVM (pneumonia virus of mice AAS87365.1), BRSV (bovine respiratory syncytial virus P22167.1), HRSV (A P03420.1 and B AAR14266.1), NDV (Newcastle disease virus AAC28374.1), AMPV (avian metapneumovirus ABQ23891.1), HPIV4 (BAA08626.1), Mumps (mumps virus P11236.1), PIV5 (P04849.1), HPIV2 (NP_598404.1), AsaPV (Atlantic salmon paramyxovirus ABW38054.1), Fer-de-Lance (Fer-de-Lance virus AAN18264.1), Sendai (AAB06281.1), HPIV1 (P12605.1), HPIV3 (AAB21447.1), BPIV3 (AZB53083.1).

The human parainfluenza viruses (HPIVs), and the related murine virus SeV, are members of the family *Paramyxoviridae*. HRSV and HMPV were also classified as paramyxoviruses until 2016 when the International Committee on the Taxonomy of Viruses (ICTV) reclassified these viruses as members of the newly created family *Pneumoviridae* [21,22]. The new species names for these viruses as defined by the 2016 reclassification are included in Table 1 and Table 2. For historical purposes and consistency with the literature, this review will use the common names. *Paramyxoviridae* genus *Respirovirus* includes HPIV1 and HPIV3 as well as their closely related animal-virus counterparts SeV and bovine PIV3 (BPIV3), respectively (Figure 1), which have been developed as vaccine platforms. The genus *Orthorubulavirus* contains human pathogens HPIV2, HPIV4, and MuV in addition to the mammalian virus PIV5, another paramyxovirus vaccine vector [23,24]. Measles virus (MeV), one of the most infectious viruses and for which there has been a safe and effective vaccine for over fifty years, is a member of the genus *Morbillivirus*.

The *Pneumoviridae* family contains HMPV from the genus *Metapneumovirus* and members of the genus *Orthopneumovirus,* including HRSV and its bovine counterpart BRSV. HRSV has been further divided into two subtypes, A and B. Paramyxoviruses and pneumoviruses are members of the order *Mononegavirales*, negative-strand RNA viruses with single stranded genomes. Other noteworthy members of this order are the families *Filoviridae* (Ebola and Marburg viruses), *Rhabdoviridae* (rabies virus and vesicular stomatitis virus, VSV), and *Bornaviridae* (Borna disease virus).

### 2.2. Genome and Virion Structure

**Figure 2:**
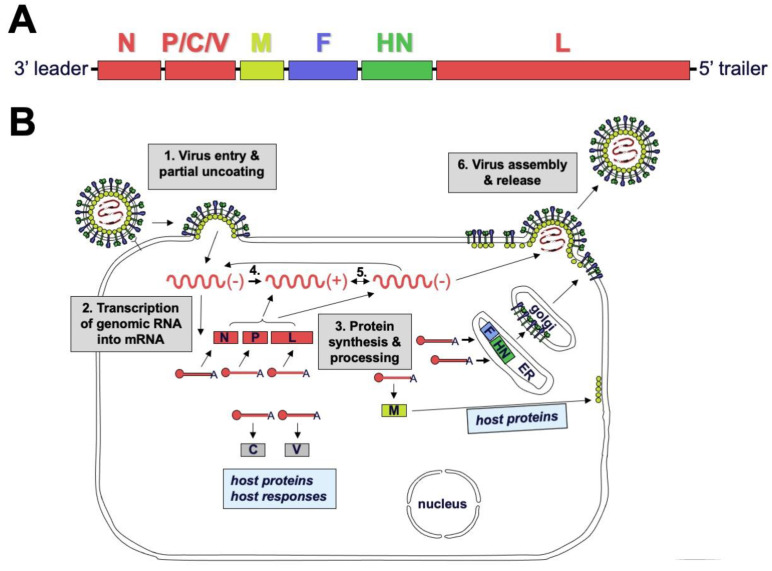
**SeV replication cycle.** (**A**) Genome structure of SeV. Polymerase complex genes nucleocapsid (N), phosphoprotein (P), and large polymerase (L) are color-coded red; matrix (M) is colored yellow; fusion (F) is colored blue; and hemagglutinin-neuraminidase (HN) is colored green. (**B**) Replication cycle of SeV. During step 1, HN binds sialic-acid-containing receptors, triggering irreversible conformational changes in the F protein that cause fusion of the viral envelope and host cell plasmid membrane. The genome and associated polymerase complexes are delivered into the cytoplasm, where they remain during replication. In step 2, the RNA-dependent RNA-polymerase transcribes viral genes serially starting from the 3′ end. In step 3, viral proteins are translated and processed. In steps 4 and 5, complementary genome is replicated and then serves as a template for replication of negative-sense genomes needed to produce progeny virions. In step 6, F and HN proteins traffic through the secretory pathway to the cell surface. The M protein associates with host cell proteins, viral ribonucleoproteins, envelope glycoprotein tails, and the plasmid membrane to help drive budding of progeny virions. HN receptor-destroying activity is needed for progeny virus release.

The genomes of paramyxoviruses and pneumoviruses are single-stranded RNA that contain all genes in tandem (Figure 2). Genes common to these two virus families encode the nucleocapsid (N) protein that encapsidates genomic viral RNA, a polymerase-associated phosphoprotein (P), a matrix (M) protein that drives virus assembly, a fusion (F) surface glycoprotein that causes membrane fusion during viral entry, and a large (L) polymerase protein [25]. Attachment proteins are named hemagglutinin-neuraminidase (HN), hemagglutinin (H), or glycoprotein (G) depending on the functions of the protein and its associated virus. For some viruses, several other structural and non-structural genes may also be expressed including alternate genes from the P gene (V, C, I, W, and Y), a small hydrophobic protein (SH), and M2-1 and M2-2 genes. In general, paramyxoviruses are spherical with a diameter of 150–350 nm but can be pleiomorphic or filamentous [25]. Pneumoviruses contain irregularly shaped spheres of 100–350 nm and filaments that are 60–200 nm in diameter and up to 10 µm in length [26,27]. Viral envelope glycoproteins project from a lipid bilayer envelope that is derived from the plasmid membrane of the host cell [28]. The SeV viral RNA, which contains 15,384 nucleotides (following the rule of six whereby the genome length is divisible by six), is bound by ~2600 N proteins, and together with 300 P and 50 L proteins, forms a helical nucleocapsid [29].

### 2.3. Replication Cycle

**Figure 3:**
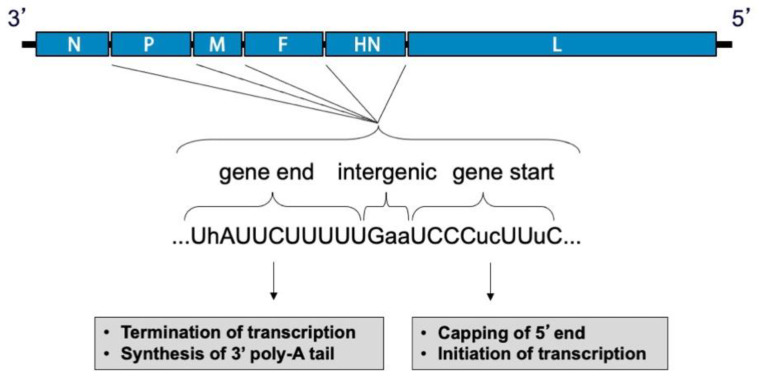
**Intergenic junctions of SeV.** Upstream of each gene is a gene start sequence that directs initiation of transcription and capping of the 5′ end of the transcript. Downstream of each gene is a gene end sequence that directs termination of transcription and synthesis of a 3′ poly-A tail. A trinucleotide sequence separates gene end from gene start between the genes. Differences in gene start sequences modulate the level of polymerase continuation of transcription versus termination.

**Figure 4:**
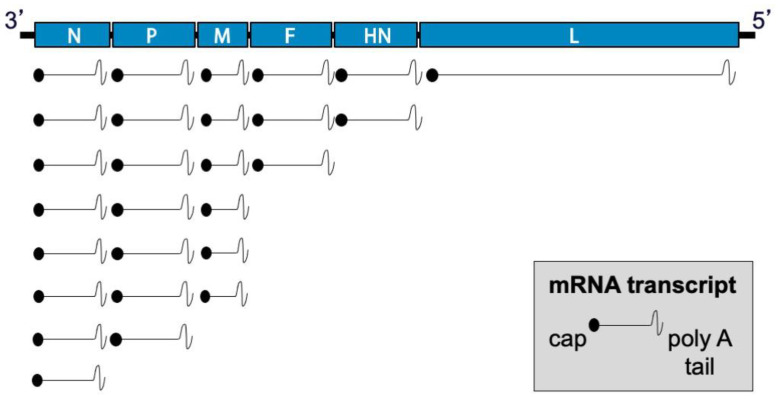
Differential transcription of SeV genes. Transcription begins at the 3′ end of the genome with the N gene. At each gene junction, the polymerase may continue to transcribe downstream genes or terminate transcription. Transcription start sequences vary between genes with the sequence upstream of the F gene resulting in the highest level of termination. As a result, the relative expression levels of F, HN, and L genes are substantially less than those of the upstream genes. Insertion of a foreign gene adds another gene junction and usually alters the ratio of gene transcripts in addition to decreasing the abundance of downstream transcripts. Positioning of foreign genes near the 3′ end of the genome causes greater virus attenuation than positioning foreign genes nearer the 5′ end.

During viral entry, the receptor-binding protein binds receptors and triggers the F protein to undergo irreversible conformational changes that cause membrane fusion, allowing delivery of the genome and polymerase complex into the cytoplasm of the host cell [30,31,32]. Replication occurs in the cytoplasm (Figure 2). Early in infection, the viral RNA-dependent RNA-polymerase (vRNAP) transcribes mRNA starting with the N gene near the 3′ end of the genome and continuing serially to the L gene at the 5′ end. Intergenic junctions between genes encode a gene end that terminates transcription and synthesizes a poly-A tail, an intergenic sequence, and a gene start that caps the 5′ end of mRNA and initiates transcription (Figure 3). At each gene junction, the vRNAP may continue or terminate, resulting in a gradient of transcripts with N being most abundant and L the least abundant (Figure 4). The gene start sequences and their propensity to allow continuation of downstream transcription may differ, and this results in a substantial drop-off of transcription between the M and F genes of SeV [33]. Insertion of a foreign gene nearer the 3′ end of the genome causes a greater reduction in viral gene expression and a larger amount of attenuation than insertion of a foreign gene nearer the 5′ end [34,35]. After sufficient expression of viral proteins, the vRNAP replicates the entire genome to a complementary strand that serves as a template for negative-sense genome that will be incorporated into progeny virions (Figure 2). Assembly and budding are directed by the M protein, which interacts with viral nucleocapsids, the inner leaflet of the plasma membrane, glycoprotein cytoplasmic tails, and cellular factors [36,37,38,39].

### 2.4. Fusion (F) Glycoprotein Structures and Structural Intermediates

The paramyxoviruses and pneumoviruses contain an F surface glycoprotein that is a Type I integral membrane protein and a structural Class I viral fusion protein [40]. High-resolution structures have been obtained for ectodomains of the prefusion forms of the F proteins from PIV5 [41], HRSV [42], HMPV [43,44], and Hendra virus [45]. The presence of the transmembrane domain and cytoplasmic tails stabilizes the prefusion form of the protein [41,46], thus cell-surface expressed F protein is expected to adopt the native structure unless heated to supraphysiological temperatures [47].

While the F proteins from different viruses share little amino-acid sequence homology, their three-dimensional folding is similar. Cleavage into F1 and F2 subunits primes the protein by conversion into a fusion-capable pre-triggered form called the prefusion conformation [48]. The prefusion structure has a mushroom-like shape. The heptad repeat B (HRB) region, adjacent to the transmembrane domain, forms a triple-stranded coiled-coil stalk. Domains II and III form a bulbous head. The fusion peptide is bound in a cleft on the side of the head and its adjacent heptad repeat A (HRA) region forms four short alpha-helices and a beta-turn-beta structure. Upon activation by receptor binding, even at low temperatures that arrest complete refolding of the F protein, the HRB triple-stranded coiled coil from the prefusion conformation dissociates [31,32,41]. Next, a prehairpin intermediate forms in which HRA springs upward into a triple-stranded coiled coil that propels the fusion peptide into the target membrane [31,32,49,50]. Finally, HRB alpha-helices bind in an antiparallel orientation into the grooves formed by the HRA coiled coil, juxtaposing the adjacent fusion peptide and transmembrane domains and providing energy to do the work of membrane fusion [31,32]. Thus, the order of conformations adopted by the F protein are: (a) native prefusion, (b) a temperature-arrested intermediate with dissociated HRB regions, (c) a prehairpin intermediate with a triple-stranded HRA coiled coil, and (d) a postfusion six-helix bundle [31]. Post-fusion structures of the F protein ectodomain have been obtained for NDV [51,52], HPIV3 [46], and HRSV [53,54].

### 2.5. Attachment Protein Structures

The HN, H, and G proteins of the paramyxoviruses and pneumoviruses are Type II integral membrane proteins. High-resolution structures have been obtained for NDV HN [55,56,57], HPIV3 HN [58], MeV H [59,60,61], PIV5 HN [62], and NiV and HeV G [63,64,65]. The paramyxovirus HN, H, and G proteins form a dimer of dimers with a rod-shaped stalk and a globular head domain that has a six-bladed beta-propeller fold that is common to sialidases. Cocrystal structures of neutralizing antibodies bound to the central conserved domain (CCD) of HRSV G have been obtained [66,67] but a high-resolution structure of the entire ectodomain has been elusive.

## 3. Past and Current Vaccine Candidates for HRSV, HMPV, and the HPIVs

Vaccine development for HRSV, HMPV, and the HPIVs has followed a long and difficult course. Among HRSV, HMPV, and the HPIVs, HRSV is the most frequent target of vaccine development because of the serious ALRTI caused by HRSV infections in human infants. In the 1960s a long-remembered study concerned a formalin-inactivated HRSV vaccine product. Unfortunately, the formalin treatment altered key epitopes on the virus surface during vaccine production, preventing the induction of neutralizing antibodies [68,69]. When vaccinated children were later naturally infected with HRSV, they fared worse than unvaccinated children. In fact, two vaccinated children died [70]. This outcome sent a shockwave through the scientific community. Since then, the fear of a repeat tragedy has hampered the clinical development of paramyxovirus and pneumovirus vaccines. The result is that six decades have passed with millions of additional lives lost due to paramyxovirus and pneumovirus infections and disease.

Since the 1960s, an astounding number of vaccines have been tested preclinically and some have progressed to clinical trials [71,72]. Strategies include the use of live-attenuated/chimeric vaccines, whole-inactivated vaccines, particle-based vaccines, subunit vaccines, nucleic acid-based vaccines, and recombinant vectors, some of which will be described below in brief. Clinical trials have involved children, pregnant women, and other adults including the elderly. An experimental human HRSV infection model also exists in which vaccines can be tested [73]. For HRSV vaccines, an online database of current research activities has been established [74]. 

A frequently used vaccine strategy has involved the attenuation of human viruses by cold-adaptation, targeted mutation, or both [75]. This strategy has proven difficult because a fine balance of attenuation must be achieved. An over-attenuated virus will not induce sufficient immunity, whereas an under-attenuated human virus may cause disease. There is the additional concern that an attenuated human virus may mutate, either during vaccine production or in a vaccinated host, to regain pathogenic potential. 

**Figure 5:**
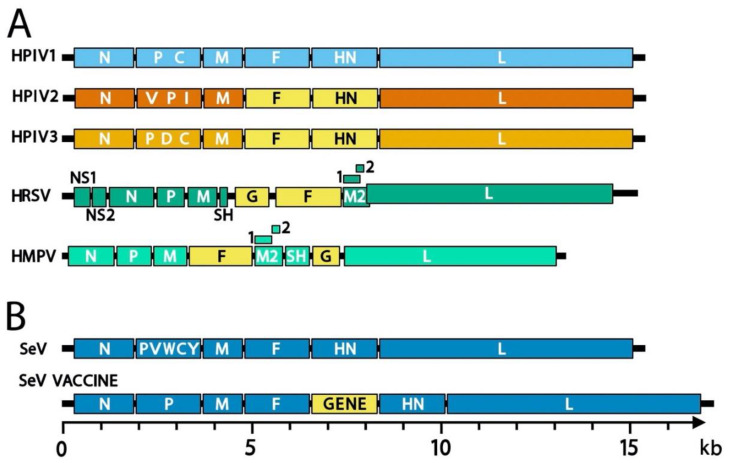
**Genome structures of representative paramyxoviruses and pneumoviruses.** (**A**) Genomes of human vaccines targeted by SeV-based vaccines. Due to high amino-acid similarity, unmodified SeV serves as a Jennerian vaccine against HPIV1. For the other human viruses, an envelope glycoprotein gene (yellow) is inserted into the SeV genome. (**B**) Sendai virus vaccine genome structures. The envelope glycoprotein gene (GENE, yellow) from a human virus may be inserted into any gene junction of the SeV genome. Most SeV-vectored vaccines studied in preclinical experiments contain a foreign gene inserted between the F and HN genes as shown. In the genomes, the 3′ leader is on the left and the 5′ trailer is on the right. Intergenic junctions (not shown) include a transcription stop, intergenic, and transcription start sequences. Genomes are drawn to scale with a scale bar at the bottom.

A separate vaccine strategy has used recombinant viruses to deliver select gene sequences to the human host for de novo expression. Both human and non-human viruses have been used as vectors, including human adenovirus 26 (now being tested by Janssen, a pharmaceutical company of Johnson and Johnson), chimpanzee adenovirus (now being tested by GlaxoSmithKline, GSK), Venezuelan equine encephalitis virus [76], modified vaccinia Ankara (MVA, now studied by Bavarian Nordic), BPIV3 [77], a BPIV3/HPIV3 chimera [78], PIV5 [23,24], and SeV (Figure 5 and described in greater detail below). Of note, the viruses BPIV3 and SeV can serve both as Jennerian vaccines for HPIV3 and hPIV1, respectively, and as vectors for recombinant gene delivery.

A number of protein-based vaccines have been tested, often with G or F proteins as their focus. The VRC, GSK, and Pfizer are each now testing HRSV F proteins, stabilized in prefusion forms (e.g., DS-Cav1) [42,44,79]. 

Particle-based vaccines have also been produced [80]. Mahdi et al. [81] performed a clinical study in pregnant women of a nanoparticle HRSV vaccine produced by Novavax. The particle was made using recombinant baculovirus that expresses the HRSV F protein. After the pregnant women gave birth, their children were monitored for ninety days for HRSV-associated, medically significant ALRTI. Infants born to vaccinated mothers experienced ALRTI reduced by 39.4% compared to placebo controls. There were mixed reviews when study data were released because the protocol’s primary endpoint was not met. Nonetheless, the results were viewed with optimism by some investigators because, for the first time, vaccination appeared to confer a degree of protection for infants against RSV.

Messenger RNA (mRNA) vaccines have come to the forefront of vaccine development in the SARS-CoV-2 field [82,83] and will assist the generation of multiple, new vaccine candidates for the paramyxovirus and pneumovirus fields [18]. Given the extraordinary effort dedicated to vaccine development for the paramyxoviruses and pneumoviruses, one can expect that new, licensed vaccine products are in sight.

## 4. Sendai Virus

SeV was discovered at Tohuko University Hospital in Sendai, Japan, in 1952 by Kuroya and colleagues after passage of a lung sample from a newborn child in mice [84]. At first, the virus was thought to be human-derived, but by 1954, Fukumi and colleagues realized that SeV was inadvertently isolated from mice [85,86]. SeV is currently recognized as a pathogen of mice, not humans, as SeV has never been known to cause human disease [87]. Outbreaks of SeV in mouse colonies worldwide have been reported including those displaying disease in mice (epizootic) [88] and others with long-term, unapparent transmission displaying little disease (enzootic) [89,90]. For enzootic strains, intranasal inoculation of large doses of SeV in large volumes that are directly aspirated into the lungs can result in substantial morbidity and mortality in susceptible strains of mice [91,92,93,94]. In contrast, contact transmission results in robust upper respiratory tract infection but limited infection and pathology in the lungs and no observable morbidity or morality in healthy mice [92,93,94]. Short-range airborne transmission initiates in the nasopharynx or trachea and can remain local or disseminate yet also causes no observable morbidity or mortality [92]. Natural infection after transmission by enzootic strains causes no apparent morbidity in mice but provides robust immunity, even upon challenge with a high (e.g., greater than 1 million infectious units) amount of virus aspirated into the lungs [93].

## 5. Sendai Virus as a Vaccine Platform

### 5.1. Sendai Virus as a Jennerian Vaccine against HPIV1

In the late 1790s, clinician Edward Jenner inoculated a young boy with a substance from cow lesions, later discovered to contain cowpox virus. When Jenner subsequently exposed the boy to smallpox, the boy was protected. The live-virus vaccine, originally isolated from bovine and naturally attenuated in humans, was later shown to elicit virus-specific B cells, T helper cells, and cytotoxic T lymphocytes that persisted for decades after a single vaccination [95,96]. Approximately two centuries after development of the Jennerian smallpox vaccine, and after a global campaign led by the World Health Organization, the human population was declared free of smallpox virus by the World Health Assembly on 8 May 1980. The smallpox vaccine campaign has been the most successful vaccination effort in history in that it completely eradicated a human disease.

During the 1990s, researchers noted amino-acid sequence and antigenic similarities between HPIV1 and SeV [97,98]. These findings underpinned the development of numerous SeV-based respiratory virus vaccines (Table 3 provides a sampling of SeV research).

Because HPIV1 and SeV are closely related phylogenetically (Figure 1), experiments were first performed to develop SeV as a Jennerian vaccine against HPIV1. The approach was supported by findings that B cell, T helper cell, and cytotoxic T lymphocytes from human blood were all cross-reactive between HPIV1 and SeV [113,114]. In a proof of principle study, an intranasal inoculation of mice with HPIV1 was shown to elicit SeV-specific antibody responses and to protect mice from challenge with SeV [115]. As another example of cross-reactivity between HPIV1 and SeV, an intranasal inoculation of mice and cotton rats with SeV rapidly elicited durable immune responses in the respiratory mucosa and protection against challenges with HPIV1 [99,116]. Intranasal inoculation of SeV in African green monkeys induced high-magnitude, durable antibody responses against both SeV and HPIV1. In two separate laboratories, SeV conferred protection in non-human primates against challenge with HPIV1 [100,101]. 

Because SeV is delivered intranasally, long-lived antibody forming cells (AFCs) and T cells are established for residence in the respiratory tract. The AFCs underlie the epithelial cells that line the respiratory airway and can secrete virus-specific IgA. IgA antibodies are uniquely suited to transcytose epithelial cells, after which they can be tethered to airway cells, providing a first line of defense against incoming pathogens. The situation is unlike that for vaccines administered intramuscularly, which more-readily induce bone marrow-resident AFCs [99,117,118,119]. Live viral vaccines are also noted, as described above, for their induction of long-term immune responses in humans [95].

The target population of an SeV vaccine is the seronegative infant. Adults and seropositive children are not the target populations for SeV because they have pre-existing immune responses toward HPIV1 that cross-react with SeV. Their responses clear the SeV vaccine so quickly that the vaccine has only minimal influence on the immune response. Nonetheless, to ensure vaccine safety, Phase I clinical studies were first initiated in adults and then progressed to SeV-seropositive 3- to 6-year-old children [102,103]. In each age group, increasing vaccine doses were tested, including 5 × 10^5^, 5 × 10^6^, and 5 × 10^7^ egg infectious doses-50 (EID_50_). As expected, replication-competent virus was not observed in these participants. Despite their pre-existing immune responses, three of nine adult participants and eight of ten children exhibited a boost in virus-specific neutralizing antibodies [102,103]. Currently, SeV is being tested in SeV-seropositive, 1- to 2-year-old children. Resultant safety data will be used to support clinical studies in the vaccine’s target population, the seronegative infant.

### 5.2. Sendai Virus-Vectored HRSV Vaccine

**Figure 6:**
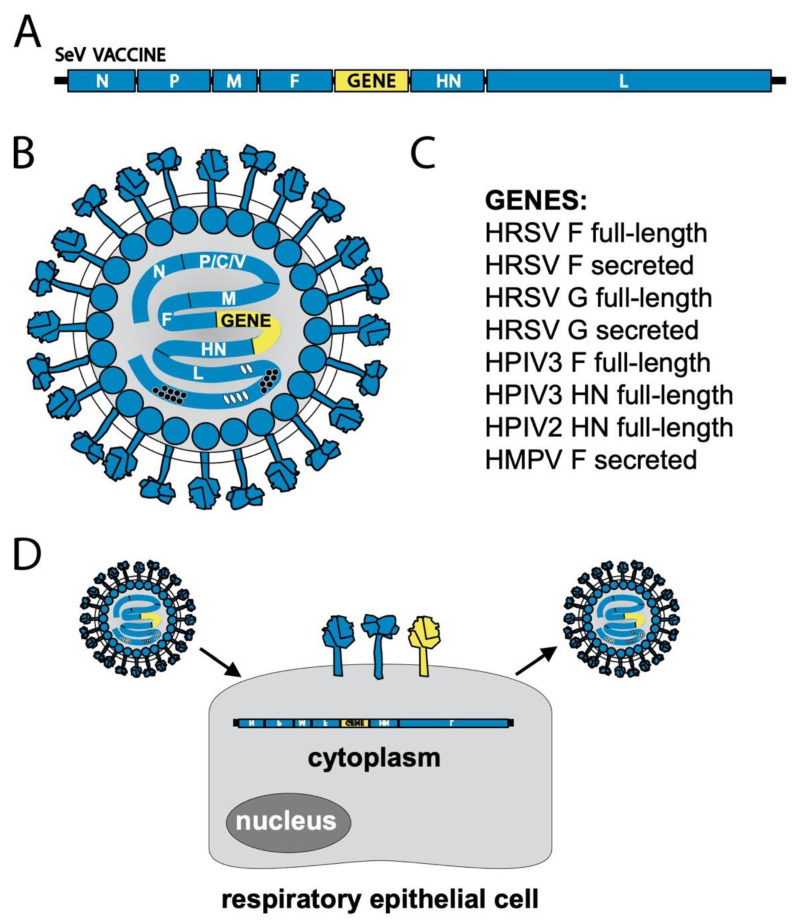
Schematic of gene delivery by SeV-vectored vaccines. (**A**) SeV vector genome structure. SeV genes are colored blue, and a foreign gene is colored yellow. Most SeV-vectored vaccines have been constructed with the inserted gene positioned between the F and HN genes, which allows for sufficient vaccine antigen expression while causing little to no attenuation of replication. (**B**) SeV virion structure. Vaccine is composed of SeV proteins (blue), SeV genome (blue), and a foreign gene (yellow). (**C**) List of genes inserted into SeV-vectored vaccines that have been tested in preclinical studies. (**D**) Schematic of vaccination. SeV-vectored vaccines are intranasally inoculated and infect respiratory epithelial cells. Replication occurs in the cytoplasm and produces both the foreign gene product (yellow) and SeV genes and genome (blue). New progeny virions are generated, but SeV replication is attenuated in species other than mice because its accessory genes are only able to counteract interferon responses in mice.

Reverse genetics systems have been available for SeV since the mid-to-late 1990′s [120,121,122]. Researchers quickly established that SeV can accommodate foreign genes of 3 kb or more [34,35,123]. This raised the possibility of using SeV as a vaccine vector (Figure 6). SeV-vectored vaccines that have full-length or secreted forms of the HRSV F or G genes inserted between the F and HN genes of SeV were generated and evaluated in preclinical animal models (Table 3) [104,105,110]. Upon infection, the SeV-vectored HRSV F and G vaccines express unmodified, full-length envelope glycoproteins on the cell surface. HRSV F or G proteins expressed from SeV were shown to be excluded from progeny SeV-vector virions. The SeV-vectored HRSV vaccines elicited robust neutralizing antibody and T cell responses in cotton rats and protected from HRSV challenge. The HRSV F gene insertion was from the A2 strain and was able to generate neutralizing antibody responses and protect from challenge by both A and B subtypes in cotton rats [105,110]. The SeV-vectored HRSV vaccines did not trigger enhanced immunopathology after challenge [104,105,110] and were shown to be effective when inoculated into cotton rats that had maternal antibodies at titers comparable to those of a 2-month-old human infant [106].

Other vectored vaccines that express unmodified, full-length HRSV F express prefusion F (Pre-F) < postfusion F (Post-F), Pre-F > Post-F, or exclusively Pre-F [124]. In the context of transmembrane-anchorless, secreted F protein constructs containing stabilizing mutations, stabilized Pre-F has been shown to enhance production of neutralizing antibodies [124]. Similarly, stabilizing proline mutations have been inserted into coronavirus spike vaccines [125,126]. It is expected that natural protein conformations including Pre-F and Post-F are expressed upon SeV-infections of mammalian cells and that protein proportions will vary as a function of mammalian cell type and time post-infection.

The SeV vaccine designated SeVRSV, which contains the full-length HRSV A2 F gene inserted into the SeV F-HN gene junction, was advanced to a non-human primate study in African green monkeys [109]. As endorsed by the Food and Drug Administration (FDA), 1 × 10^6^ EID_50_ of the vaccine was administered both intranasally and intratracheally, and the African green monkeys were challenged with 1.4 × 10^6^ PFU of the A2 strain of HRSV 28 days after vaccination. Vaccination stimulated production of HRSV-specific binding and neutralizing antibodies and protected against HRSV challenge without inducing immunopathology [109]. Subsequently, the SeV-vectored HRSV-F vaccine was advanced to human clinical trials in adults who were expected to be HRSV- and HPIV1/SeV-seropositive [127]. Again, as expected because of pre-existing immunity in adults, vaccine was quickly cleared. The recombinant vaccine genome was detected only transiently by PCR (tests of replication-competent SeVRSV were not performed). Furthermore, as expected, boosts of pre-existing immune responses were rare. Importantly, the vaccine was well tolerated, inducing only mild to moderate reactions that were also observed in the placebo group. These results encourage progression of the SeV-vectored HRSV vaccine toward clinical studies in seronegative infants. Partnerships with the Serum Institute of India and the National Institutes of Health are ongoing to advance the SeVRSV vaccine through clinical trials.

### 5.3. Sendai Virus-Vectored HPIV3 Vaccine

SeV-vectored vaccines with the HPIV3 F or HN gene inserted between the F and HN genes of the SeV genome were generated and tested preclinically in cotton rats [107]. The vaccines were shown to express the inserted HPIV3 gene in infected Hep-2 cells. Vaccination of cotton rats resulted in the production of virus-specific binding antibodies, neutralizing antibodies, and interferon-gamma-producing T cells. Vaccination protected against the homotypic HPIV3 strain (C243) and an HPIV3 clinical isolate. Additional studies were completed on the HPIV3 F vaccine, varying the site of foreign antigen insertion (P-M or F-HN gene junction) or inoculated vaccine dose (200 or 2,000,000 PFU) [111]. While the P-M insertion caused mild attenuation in LLC-MK2 cells and cotton rats, it grew similarly to the F-HN construct in NHBE cells and yielded high levels of virus-specific neutralizing antibodies in cotton rat sera. Lower-dose vaccination resulted in only modest decreases in vaccine replication and serum antibody responses. All four vaccine combinations (two viruses and two doses) elicited complete protection from HPIV3 challenge in cotton rats, demonstrating the versatility of the SeV vaccine platform. 

### 5.4. Sendai Virus-Vectored HPIV2 Vaccine and SeV-Vectored Vaccine Cocktails

Two SeV-vectored HPIV2 vaccines were generated by inserting the HPIV2 F or HN gene into the SeV F-HN gene junction [108]. Vaccination of cotton rats elicited serum neutralizing antibodies against the homotypic strain and heterotypic HPIV2 clinical isolates. Antibodies were durable for at least nine months after vaccination. The SeV-vectored vaccines also yielded complete protection from HPIV2 growth in the cotton rat lungs after challenge, even 9 and 11 months after vaccination. Simultaneous intranasal inoculation in cotton rats of a three-component cocktail of recombinant SeVs expressing HPIV2, HPIV3, and HRSV antigens yielded complete protection from challenge by HPIV1, HPIV2, HPIV3, and HRSV [108]. This suggests a cocktail of SeV-vectored vaccines could be administered early in childhood to target multiple respiratory pathogens.

### 5.5. Sendai Virus-Vectored HMPV Vaccine

An SeV-vectored HMPV was generated that had a truncated HMPV F gene inserted between the F and HN genes of the SeV genome [112]. As with the other SeV-vectored vaccines, intranasal vaccination of cotton rats elicited binding and neutralizing serum antibody responses and protected from challenge with the target virus, in this case HMPV [112,128].

## 6. Immunocompetence and the Vaccinated Host

A successful vaccine program depends on the immunocompetence of host populations. Even when vaccines prove clinically efficacious and advance to licensure, a subset of humans will not respond well. If an individual is receiving immunosuppressive drugs (e.g., for organ transplantation or treatments for allergy/autoimmune disease), it is understood that vaccine-induced immune responses may be sub-optimal. Less well recognized are the weak vaccine-induced immune responses among individuals who are vitamin-deficient or obese. Vitamin A deficiencies often go unnoticed in wealthy countries because they are presumed to affect the developing world exclusively. In fact, wealthy countries encompass low-income communities where there is little access to vitamin-rich foods [129] and frequent cases of vitamin deficiencies [130]. In children, vitamin A deficiencies/insufficiencies associate with poor immune responses toward vaccines [131]. Individuals with obesity, although apparently vitamin-replete based on blood tests, can suffer from low vitamin A levels in tissues including the lung [132] and from poor responses to vaccines [133,134].

In animal models of vitamin A deficiencies and obesity, supplementation with vitamin A at the time of vaccination improved immune responses toward vaccines [134,135,136,137,138]. In a clinical study, vitamin supplementation of children who had low baseline levels of vitamins A and D also significantly improved responses toward an influenza vaccine [131].

Supplementation programs have had positive outcomes in geographical areas where nutritional deficiencies are endemic [139]. However, because humans are a highly heterogeneous population, a one-size-fits-all vitamin supplementation program does not suffice [131]. This is due, in part, to the cross-regulation between vitamin A, a nuclear receptor ligand, and related ligands including vitamin D, sex hormones, and fat [140,141,142,143,144,145,146]. To assist the design of vitamin supplementation programs that may provide overall clinical benefit at the population level, a better understanding is needed of (i) baseline levels of nuclear receptor ligands in vaccine recipients and (ii) nuclear receptor ligand cross-regulatory capacities.

## 7. Outlook

Based on positive results from passive transfer studies, we know that immune effectors can protect humans from serious paramyxovirus and pneumovirus infections. Vaccines must now be advanced to ensure that virus-specific lymphocytes are activated in infants before a first virus exposure occurs. The SeV-based vaccines are attractive candidates for this purpose because they activate virus-specific lymphocytes and ensure long-term residence of immune cells in the respiratory tract. The COVID-19 pandemic has taught us that vaccine development can be rapid in response to an immediate need. Severe diseases caused by HRSV, HMPV, and HPIVs define another immediate need and encourage a call-to-action for rapid vaccine development. Other paramyxovirus vectors such as PIV5 and measles virus have been engineered to express glycoproteins from positive-strand viruses such as alphaviruses and coronaviruses [147,148,149]. SeV vaccines targeting positive-strand RNA viruses could also be produced and tested. Because of SeV’s cross-reactivity with HPIV1 and the immune responses generated in humans toward SeV by HPIV1 exposures, Sendai-vectored vaccines are best targeted to the pediatric arena. In this scenario, SeV-vectored vaccines have many attractive features, including high-level production in embryonated hen eggs or mammalian cell cultures, natural attenuation in humans, demonstrated safety in humans, robust induction of mucosal and systemic B- and T-cell responses, and potent and durable immunogenicity. With combined efforts from government agencies, pharmaceutical companies, and research institutes, the outlook for an upcoming vaccine success is good.

## Figures and Tables

**Table 1:** Nomenclature of several viruses from Family Paramyxoviridae.

Common Name	Species	Genus
Human parainfluenza virus 1 (HPIV1)	*Human respirovirus 1*	*Respirovirus*
Human parainfluenza virus 3 (HPIV3)	*Human respirovirus 3*	
Sendai virus (SeV)	*Murine respirovirus*	
Human parainfluenza virus 2 (HPIV2)	*Human orthorubulavirus 2*	*Orthorubulavirus*
Human parainfluenza virus 4 (HPIV4)	*Human orthorubulavirus 4*	
Parainfluenza virus 5 (PIV5)	Mammalian orthorubulavirus 5	
Mumps virus (MuV)	Mumps orthorubulavirus	
Canine distemper virus (CDV)	*Canine morbillivirus*	*Morbillivirus*
Measles virus (MeV)	*Measles morbillivirus*	
Newcastle disease virus (NDV)	*Avian avulavirus 1*	*Orthoavulavirus*
Hendra virus (HeV)	*Hendra henipavirus*	*Henipavirus*
Nipah virus (NiV)	*Nipah henipavirus*	

**Table 2:** Nomenclature of several viruses from Family *Pneumoviridae*.

Common Name	Species	Genus
Human metapneumovirus (HMPV)	*Human metapneumovirus*	*Metapneumovirus*
Bovine respiratory syncytial virus (BRSV)	*Bovine orthopneumovirus*	*Orthopneumovirus*
Human respiratory syncytial virus (HRSV)	*Human orthopneumovirus*	

**Table 3:** Examples of research with SeV-based respiratory virus vaccines.

Vaccine Name	Inserted Antigen	Insertion Site	Host	Reference
Sendai virus	None	None	cotton rats	[99]
Sendai virus	None	None	African green monkeys (AGM)	[100]
Sendai virus	None	None	AGM, chimpanzees	[101]
Sendai virus	None	None	human (adults/3–6 y.o.)	[102,103]
rSV-RSV-G	RSV A2 G	F-HN	cotton rats	[104,105]
rSV-RSV-F (SeVRSV)	RSV A2 F	F-HN	cotton rats	[105,106,107,108]
rSV-RSV-F (SeVRSV)	RSV A2 F	F-HN	AGM	[109]
rSV-RSV-F (SeVRSV)	RSV A2 F	F-HN	human adults	[103]
rSV-RSV-Fs	RSV A2 F (secreted)	F-HN	cotton rats	[110]
rSV-HPIV3-HN	HPIV3 C243 HN	F-HN	cotton rats	[107,108]
rSV-HPIV3-F	HPIV3 C243 F	F-HN	cotton rats	[107,111]
rSV-HPIV3-F(P-M)	HPIV3 C243 F	P-M	cotton rats	[111]
rSV-HPIV2-HN	HPIV2 VR92 HN	F-HN	cotton rats	[108]
rSV-HPIV2-F	HPIV2 VR92 F	F-HN	cotton rats	[108]
rSV-HMPV-Fs	HMPV CAN00-16 F (secreted)	F-HN	cotton rats	[112]
